# The complete mitochondrial genome of *Trichoderma simmonsii* (Hypocreales: Hypocreaceae) from the Southern Coast of Korea

**DOI:** 10.1080/23802359.2022.2060766

**Published:** 2022-04-08

**Authors:** Dawoon Chung, Yong Min Kwon, Youngik Yang

**Affiliations:** a National Marine Biodiversity Institute of Korea, Seocheon-gun, South Korea

**Keywords:** *Trichoderma simmonsii*, complete mitochondrial genome

## Abstract

Fungal species in the genus *Trichoderma* are widely used for industrial enzyme production and as biocontrol agents. In this study, we report the complete mitochondrial genome of a marine-derived *Trichoderma simmonsii* strain GH-Sj1, which belongs to the Harzianum clade of *Trichoderma*. GH-Sj1 was isolated from an edible sea alga *Saccharina japonica* collected from the southern coast of Korea. This newly assembled circular molecule is 28,668 bp in length and consists of 15 protein-coding genes, 26 transfer RNA genes, and two ribosomal RNA genes. Phylogenetic analysis using the maximum likelihood method shows that *T. simmonsii* GH-Sj1 is closely related to *Trichoderma harzianum* and *Trichoderma lixii*. To the best of our knowledge, this is the first characterization of a marine-derived mitogenome within the genus *Trichoderma*.

Members of the genus *Trichoderma* are known to produce commercial enzymes including cellulase and chitinase (di Cologna et al. 2018). In addition, they are utilized in agriculture to control fungal pathogens and promote plant growth (Zin and Badaluddin 2020). *Trichoderma simmonsii* Chaverri, Rocha, Samuels, Degenkolb, and Jaklitsch 2015, in the family Hypocreaceae, was first identified in the United States and has been reported in Europe and East Asia (Chaverri et al. 2015). Unlike other *T. simmonsii* strains which were isolated mostly from decaying bark and decorticated wood, the specimen for this study (strain GH-Sj1) was isolated from *Saccharina japonica*, a very well-known edible sea algae called Kombu. It was collected from Gul-Hang Quay at Sacheon, Gyeongsangnamdo Province, Republic of Korea (34.55′43.5″N, 128.03′24.8″E), following the Guideline for Investigations in Marine Life produced by National Marine Biodiversity Institute of Korea (MABIK). Genomic DNA (gDNA) extraction was performed by a phenol-chloroform method, and the fungal isolate and gDNA were deposited at MABIK (https://www.mabik.re.kr, Dawoon Chung, and dwchung@mabik.re.kr) under voucher number FU00001116.

Paired-end reads were generated using Illumina Hiseq NovaSeq (Illumina, San Diego, CA) and assembled using NOVOPlasty v3.6 (Dierckxsens et al. 2017), where the complete mitochondrial genome of *Trichoderma lixii* (NCBI accession NC_052832) was used as the seed sequence. The assembled mitogenome was annotated on Geneious Prime v2021.03 (Biomatters, Auckland, New Zealand) with the genome of *Trichoderma hamatum* (NC_036144) as reference. The complete mitochondrial genome of *T. simmonsii* GH-Sj1 (GenBank accession MZ292901) is 28,668 bp long, where GC content is 27.6% and the overall base composition of A, C, G, and T is 36.1%, 12.5%, 15.1%, and 36.3%, respectively. The mitogenome harbors 15 protein-coding genes, which start with ATG except for *rps3* of ATA. The stop codon for all genes is TAA.

There are 26 transfer RNA (tRNA) genes with lengths ranging from 70 bp to 87 bp. Of these 26 tRNA genes, tRNA^Met^ has three copies, while tRNA^Ser^, tRNA^Phe^, tRNA^Gln^, and tRNA^Arg^ have two copies. Other 15 tRNAs have a single copy. A group of 12 tRNA genes lie between *rps3* and *nad2*. The mitogenome also contains two ribosomal RNA (rRNA), small subunit rRNA (SSU) and large subunit rRNA (LSU), where the lengths of SSU and LSU are 1489 bp and 4709 bp, respectively.

A phylogenetic analysis was performed with mitogenomes of 12 fungal species belonging to the order Hypocreales. The mitochondrial genome of *Clonostachys rosea* from the family Bionectriaceae was selected as an out-group. A maximum likelihood (ML) tree was constructed using RAxML v8.2.10 (Stamatakis 2014) from 14 single copy orthologous genes with GAMMA-JTT protein substitution model and 100 bootstrapping options, which placed *T. simmonsii* GH-Sj1 among Hypocreaceae with high bootstrap support values between 72% and 100% (Figure 1). In addition, *T. simmonsii* GH-Sj1 formed monophyletic clade with *T. harzianum*, *T. lixii* and *T. reesei*. In conclusion, the complete mitogenome of *T. simmonsii* GH-Sj1 will provide an important genetic resource and further understanding of mitochondrial evolution in fungi.

**Figure 1.**
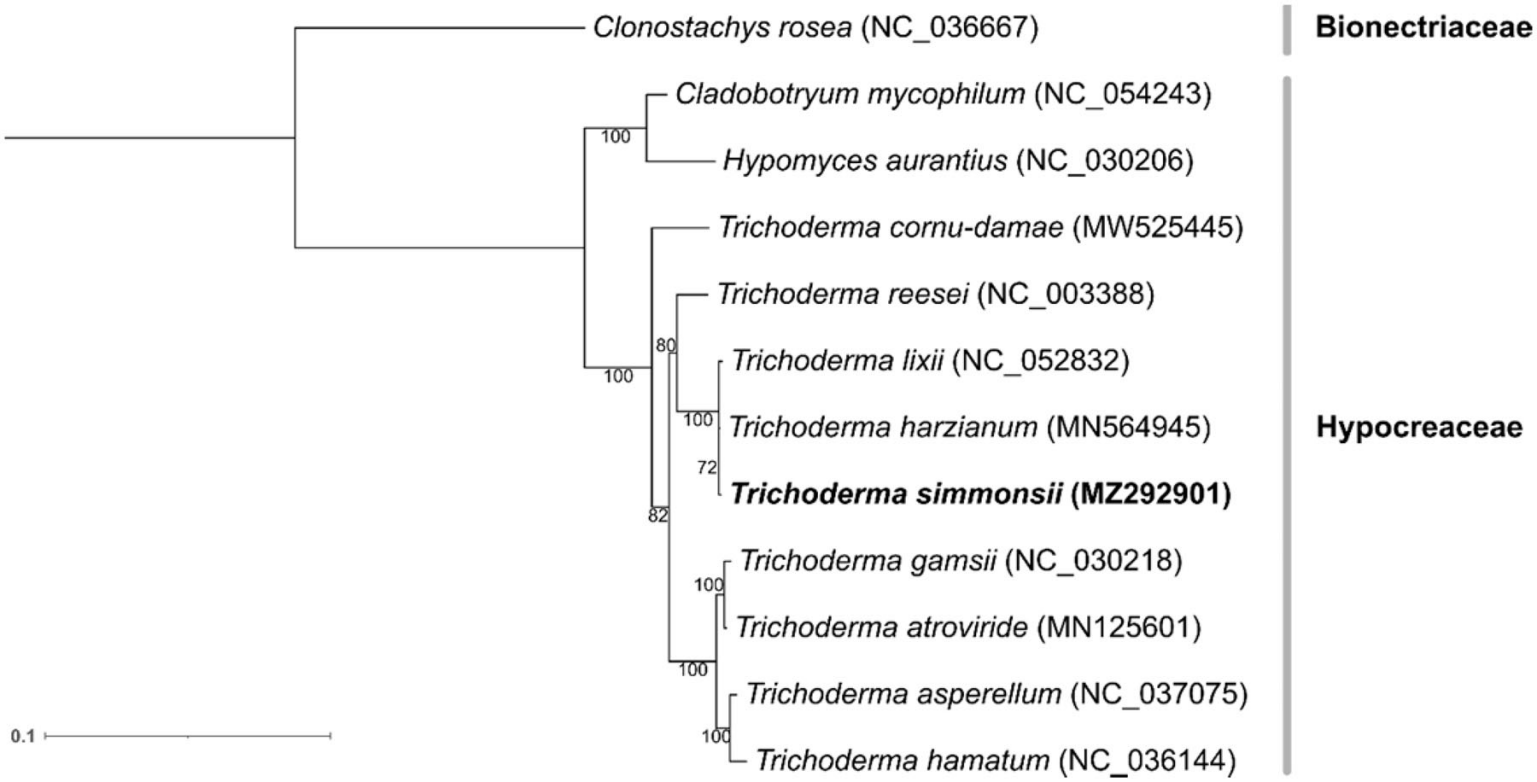
Maximum-likelihood (ML) phylogeny of 12 Hypocreales mitogenomes. *Trichoderma simmonsii* GH-Sj1 is shown in bold. Protein-coding genes of the mitogenomes were aligned by MUSCLE v3.8.31 (Edgar 2004) wherein 14 single copy orthologous genes were identified. An ML tree was then generated using RAxML v8.2.10 (Dierckxsens et al. 2017) from the single orthologous genes with GAMMA-JTT protein substitution model supported by 100 bootstrap replicates.

## Ethical approval

The marine samples collected and used for this study do not involve any marine organisms under protection determined by Ordinance of the Ministry of Oceans and Fisheries in Republic of Korea. Therefore, our study was exempted from the ethical approval and did not need any permissions to carry it out.

## Authors’ contributions

YMK and DC isolated the sample. DC and YY performed the experiment. YY performed mitogenome analyses. All authors wrote the manuscript. All authors have read and accepted the final manuscript.

## Data Availability

The genome sequence data that support the findings of this study are openly available in GenBank of NCBI at [https://www.ncbi.nlm.nih.gov] (https://www.ncbi.nlm.nih.gov/) under the accession no. MZ292901. The associated NCBI’s BioProject, SRA, and Bio-Sample numbers are PRJNA645793, SRR14597879, and SAMN15516371, respectively.
